# Kir2.1 Interactome Mapping Uncovers PKP4 as a Modulator of the Kir2.1-Regulated Inward Rectifier Potassium Currents

**DOI:** 10.1074/mcp.RA120.002071

**Published:** 2020-11-25

**Authors:** Sung-Soo Park, Daniela Ponce-Balbuena, Rork Kuick, Guadalupe Guerrero-Serna, Justin Yoon, Dattatreya Mellacheruvu, Kevin P. Conlon, Venkatesha Basrur, Alexey I. Nesvizhskii, José Jalife, Jean-François Rual

**Affiliations:** 1 Department of Pathology, University of Michigan Medical School, Ann Arbor, Michigan, USA; 2 Department of Internal Medicine and Center for Arrhythmia Research, University of Michigan, Ann Arbor, Michigan, USA; 3 Department of Biostatistics, School of Public Health, University of Michigan, Ann Arbor, Michigan, USA; 4 Department of Computational Medicine & Bioinformatics, University of Michigan, Ann Arbor, Michigan, USA; 5 Fundación Centro Nacional de Investigaciones Cardiovasculares Carlos III, Madrid, Spain; 6 Centro de Investigación Biomédica en Red de Enfermedades Cardiovasculares, Madrid, Spain

**Keywords:** Protein-protein interactions*, cardiovascular disease, cardiovascular function or biology, mass spectrometry, macromolecular complex analysis, BioID, cardiomyopathy, inward rectifier potassium current, Kir2.1, PKP4

## Abstract

Kir2.1, a strong inward rectifier potassium channel encoded by the *KCNJ2* gene, is a key regulator of the resting membrane potential of the cardiomyocyte and plays an important role in controlling ventricular excitation and action potential duration in the human heart. Mutations in *KCNJ2* result in inheritable cardiac diseases in humans, *e.g.* the type-1 Andersen-Tawil syndrome (ATS1). Understanding the molecular mechanisms that govern the regulation of inward rectifier potassium currents by Kir2.1 in both normal and disease contexts should help uncover novel targets for therapeutic intervention in ATS1 and other Kir2.1-associated channelopathies. The information available to date on protein-protein interactions involving Kir2.1 channels remains limited. Additional efforts are necessary to provide a comprehensive map of the Kir2.1 interactome. Here we describe the generation of a comprehensive map of the Kir2.1 interactome using the proximity-labeling approach BioID. Most of the 218 high-confidence Kir2.1 channel interactions we identified are novel and encompass various molecular mechanisms of Kir2.1 function, ranging from intracellular trafficking to cross-talk with the insulin-like growth factor receptor signaling pathway, as well as lysosomal degradation. Our map also explores the variations in the interactome profiles of Kir2.1^WT^*versus* Kir2.1^Δ314-315^, a trafficking deficient ATS1 mutant, thus uncovering molecular mechanisms whose malfunctions may underlie ATS1 disease. Finally, using patch-clamp analysis, we validate the functional relevance of PKP4, one of our top BioID interactors, to the modulation of Kir2.1-controlled inward rectifier potassium currents. Our results validate the power of our BioID approach in identifying functionally relevant Kir2.1 interactors and underline the value of our Kir2.1 interactome as a repository for numerous novel biological hypotheses on Kir2.1 and Kir2.1-associated diseases.

## Teaser

A comprehensive map of the Kir2.1 interactome was generated using the proximity-labeling approach BioID. The map encompasses 218 interactions, the vast majority of which are novel, and explores the variations in the interactome profiles of Kir2.1^WT^ versus Kir2.1^Δ314-315^, a trafficking deficient ATS1 mutant, thus uncovering molecular mechanisms whose malfunctions may underlie ATS1 disease. PKP4, one of the BioID interactors, is validated as a modulator of Kir2.1-controlled inward rectifier potassium currents.

## Graphical Abstract

**Figure:**
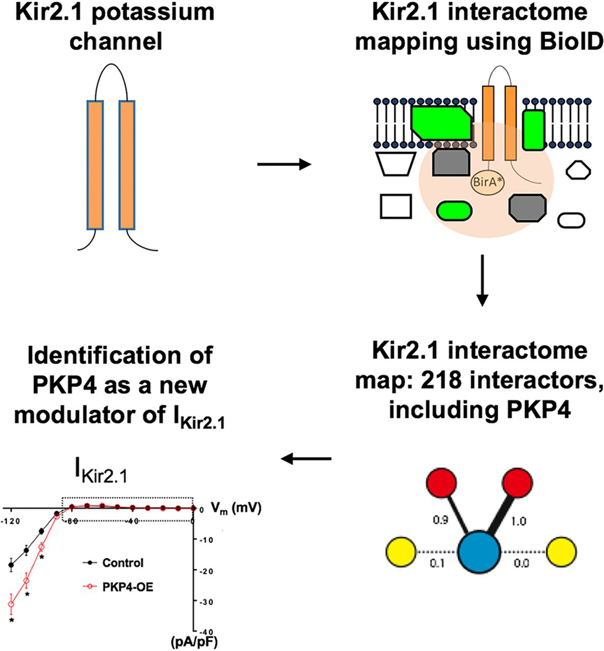

**Highlights**

• Generation using BioID of a map of the Kir2.1 interactome with 218 interactions.
• Identification of Kir2.1^WT^- versus Kir2.1^Δ314-315^-preferred interactors.
• Identification of the desmosome protein PKP4 as a new modulator of I_Kir2.1_ currents.

The strong inward rectifier potassium channel Kir2.1 is a key regulator of the resting membrane potential of the cardiomyocyte and plays an important role in controlling ventricular excitation and action potential duration in the human heart (1, 2, 3). Both loss and gain of function mutations in *KCNJ2*, the gene encoding Kir2.1, result in inheritable cardiac ion channel diseases. For example, several *KCNJ2* loss of function mutations are associated with the inheritable type-1 Andersen-Tawil syndrome (ATS1), also known as long QT syndrome type 7, which predisposes patients to cardiac arrhythmias and sudden death (4, 5). On the other hand, *KCNJ2* gain-of function mutations give rise to the type-3 variant of the short QT syndrome, which also results in increased risk of sudden cardiac death (6). Understanding the molecular mechanisms that govern the regulation of inward rectifier potassium currents by Kir2.1 in both normal and disease contexts should help uncover novel targets for therapeutic intervention in ATS1 and other Kir2.1-associated channelopathies.

Over the last 20 years, analyses of the Kir2.1 channelosome using genetic, pharmacological, and molecular approaches have greatly contributed to our understanding of its function and its role in cardiac diseases (3, 7, 8). Expression, trafficking, localization, and function of Kir2.1 are regulated by its interactions with multiple proteins. To date, 24 putative Kir2.1 interactors have been identified, out of which 16 are high-confidence Kir2.1 interactors (supplemental Table S1). For example, Leonoudakis *et al.* showed that the PDZ-binding motif of Kir2.1 interacts with the SAP97, CASK and LIN7C proteins, members of the membrane-associated guanylate kinase (MAGUK) family, which work both as scaffolding proteins for large macromolecular structure and as trafficking regulators (9, 10, 11). Recently, we and others demonstrated that the Kir2.1 channel and the voltage-gate sodium channel, Nav1.5, belong to common multiprotein channelosomes, enabling one to regulate the other's expression (12, 13, 14, 15). It has become apparent that ion channel proteins do not function in isolation but are part of large, multi-protein complexes, comprising not only the ion channels and their auxiliary subunits, but also components of the cytoskeleton, regulatory post-translation modification enzymes, trafficking proteins, extracellular matrix proteins, and even other ion channels (3, 7, 8). Notwithstanding the significance of the aforementioned biochemical studies, the information available on protein-protein interactions involving Kir2.1 channels, with only 16 high-confidence Kir2.1 interactors, remains limited. Additional efforts are necessary to provide a comprehensive map of the Kir2.1 interactome.

The relatively low number of known Kir2.1 interactors described to date in the literature can be attributed to its hydrophobic nature and the technical challenges associated with its biochemical manipulation. Similarly, some members of the protein complexes associated with Kir2.1 may mediate weak, transient interactions that are lost during “standard” biochemical affinity purification in the various lysis, wash, and elution steps. BioID, a cutting-edge proximity-labeling technology that is particularly well-suited to mapping protein interactions for low-solubility proteins, overcomes many of the above barriers imposed by conventional screening methods (16, 17, 18). BioID is based on the fusion of a promiscuous *E. coli* biotin protein ligase (BirA*) to a bait protein (17). On expression of the BirA*-bait fusion protein in cell and subsequent addition of biotin, proteins that are near-neighbors of the fusion protein are biotinylated in a proximity-dependent manner. Biotinylated proteins are then isolated by affinity capture and identified by MS (MS), thus uncovering the BioID interactome of the bait protein under investigation (17). A key advantage of this proteomic approach resides in the fact that the bait, *e.g.* the low-solubility channelosome protein Kir2.1, does not need to be biochemically purified in native complex and that transient interactions can be captured (16, 17, 18).

Here, we present the generation of a comprehensive map of the Kir2.1 interactome using BioID. Bait proteins used in our BioID proteomic screens include not only Kir2.1^WT^ but also Kir2.1^Δ314-315^, an ATS1-associated mutation, which blocks Kir2.1 Golgi export (4, 19, 20). Thus, beyond interactome mapping, our BioID experiments also aim to capture the differences between the protein interaction profiles of Kir2.1^WT^ *versus* Kir2.1^Δ314-315^ proteins, and to uncover the molecular mechanisms whose malfunctions underlie ATS1 disease. A total of 218 high-confidence Kir2.1 BioID interactors were identified, including 75 Kir2.1^WT^-preferred interactors, 66 Kir2.1^Δ314-315^ mutant-preferred interactors and 77 proteins that interact with both WT and mutant Kir2.1 proteins. Finally, we present patch-clamp analyses of one of our top BioID interactors, PKP4, which validate its functional relevance to modulation of Kir2.1-regulated inward rectifier potassium currents.

## EXPERIMENTAL PROCEDURES

##### Plasmids and Antibodies

For the transmembrane protein control (TM-CTRL), the amino acid sequence IIFRTLFGSLVFAIFLILMIN of the *Saccharomyces cerevisiae* P25353 protein transmembrane domain, which has no homology to any human proteins, was selected and a humanized codon sequence was synthesized (supplemental Table S2). The protein-encoding Open Reading Frames (ORFs) of *Kir2.1^WT^* (NM_000891.2), *Kir2.1*^Δ*314-315*^, and TM-CTRL were first cloned by Gateway recombination cloning from the cDNA plasmids into the Gateway Donor vector pDONR223 to generate Entry clones, as previously described (21) (primer sequences are shown in supplemental Table S2). The Gateway clone of *PKP4* (EL733946) was obtained from the Center for Cancer Systems Biology (CCSB, Harvard Medical School, Boston) human ORFeome collection (21). Protein-encoding ORF/s were then subcloned from the Entry clones into the Gateway Destination vectors pDEST-pcDNA5-FRT-BirA*-FLAG-N-term and pDEST-pcDNA3-HA (18, 22). A target sequence of *PKP4* for CRISPR/Cas9 (supplemental Table S2) was selected using CHOPCHOP web tool (23) and cloned into pX459 (Addgene, Watertown, MA, 62988), as previously described (24). The following primary and secondary antibodies were used in the Western blot analyses: FLAG (Sigma, Kawasaki, Kanagawa, Japan, A8592) and goat α-rabbit IgG (Cell Signaling, Danvers, MA, 7074).

##### Cell Culture

Flp-In T-REx 293 cells (ThermoFisher Scientific, Waltham, MA, R78007) were maintained following the manufacturer's guideline and were transfected with the Gateway expression vectors (pcDNA5-FRT-BirA*-FLAG-N-term) of Kir2.1^WT^, Kir2.1^Δ314-315^, and TM-CTRL using polyethylenimine (Polysciences, Warrington, PA, 23966-2) (co-transfection with pOG44) as previously described (25). Stable cells were selected using 200 µg/ml hygromycin B (Invitrogen, Carlsbad, CA, 10687010). HEK293T cells were cultivated in DMEM (Invitrogen, 11995) medium supplemented with 10% FBS (Sigma, F0926) and penicillin/streptomycin (Invitrogen, 15140) and were transfected with protein expression vectors using PEI.

##### Affinity Purification of Biotinylated Proteins

BioID experiments were performed as previously described (18) with some minor modifications. Triplicate BioID experiments were performed for each of the bait proteins: BirA*-Kir2.1^WT^, BirA*-Kir2.1^Δ314-315^ and BirA*-TM-CTRL. Cells were incubated for 24 h with 10 µg/ml tetracycline and 50 μm biotin. After washing with cold PBS, cells (*n* = 3 × 10^7^) were lysed for 30 min at 4°C with lysis buffer [50 mm Tris pH 7.2, 150 mm NaCl, 10% glycerol, 1% Nonidet P-40, 0.5% sodium deoxycholate, 0.2% SDS, protease inhibitor (Roche, Basel, Switzerland, 05 056 489 001), phosphatase inhibitor (Sigma, P0044), and Benzonase (Sigma, E1014)]. The cleared cell lysates were incubated with 30 μL of streptavidin bead slurry (ThermoFisher Scientific, 20361) for 3 h at 4°C. The beads were washed with washing buffer (50 mm Tris pH 7.2, 150 mm NaCl, 10% glycerol, 1% Nonidet P-40, 0.5% sodium deoxycholate, 0.2% SDS) four times and then with rinse buffer (50 mm Tris pH 7.2, 150 mm NaCl, 0.5% Nonidet P-40) three times. The beads were washed with fresh 50 mm NH_4_HCO_3_ three times and stored at −80°C.

##### Protein Identification by Mass Spectrometry and BioID Hit Selection

The samples were analyzed in the Proteomics Resource Facility of the Department of Pathology at the University of Michigan for protein identification. Briefly, upon reduction (10 mm DTT) and alkylation (65 mm 2-chloroacetamide) of the cysteines, proteins were digested overnight with 500 ng of sequencing grade, modified trypsin (Promega, Madison, WI, V5111). Resulting peptides were resolved on a nano-capillary reverse phase column (Acclaim PepMap C18, 2 μm, 50 cm, ThermoFisher Scientific, ES803) using 0.1% formic acid/acetonitrile (ACN) gradient at 300 nL/minute (2–25% ACN in 108 min, 25–40% in 20 min, followed by a 90% ACN wash for 10 min and a further 30 min' re-equilibration with 2% ACN) and directly introduced into Orbitrap Fusion Tribrid Mass Spectrometer (ThermoFisher Scientific). MS1 scans were acquired at 120k resolution. Data-dependent high-energy C-trap dissociation MS/MS spectra were acquired with top speed option (3 s) following each MS1 scan (relative CE ∼32%). Proteins were identified by searching the data against *Homosapiens* database containing both canonical and isoform protein entries (SwissProt v2016-11-30; total number of entries: 42054) using Proteome Discoverer (v2.1, ThermoFisher Scientific, June 2016) with enzyme specificity for trypsin. Search parameters included MS1 mass tolerance of 10 ppm and fragment tolerance of 0.1 Da; two missed cleavages were allowed; carbamidomethylation of cysteine was considered fixed modification and oxidation of methionine, deamidation of asparagine and glutamine were considered as variable modifications. False discovery rate (FDR) was determined using target-decoy strategy and proteins/peptides with a FDR of ≤1% were retained. A stringent set of criteria were used for the selection of the high-confidence Kir2.1 BioID hits: (1) average SpC (spectral counts) in either the Kir2.1^WT^ or the Kir2.1^Δ314-315^ BioID experiment > 10; (2) SAINT probability (27, 28) > 0.9; and (3) primary fold change FC-A score (28) > 7. The data obtained with the TM-CTRL bait were used as negative controls for the calculations. One of the high-confidence Kir2.1 BioID interactors has been identified with a single peptide, *i.e.* NACA2. The spectra annotation for NACA2 is provided in the supplemental Fig. S1.

##### Kir2.1^WT^ versus Kir2.1^Δ314-315^ Interactome Profiling

We classified the BioID hits as Kir2.1^WT^-preferred or Kir2.1^Δ314-315^ -preferred interactors based on the normalized Kir2.1^WT/Δ314-315^ SpC ratio “*R* “. For a given protein, “*R* “ represents the ratio of the SpC observed for this protein in the Kir2.1^WT^ and Kir2.1^Δ314-315^ BioID experiments. Specifically, *R* was calculated as follows. First, the number of SpC observed for a given protein in a given triplicate Kir2.1^WT^ or Kir2.1^Δ314-315^ BioID experiment was normalized to the Kir2.1^WT^ BioID experiment #1 by dividing SpC by the ratio of the Kir2.1 SpC observed in this given experiment to the Kir2.1 SpC observed in the Kir2.1^WT^ BioID experiment #1 (supplemental Table S3, columns X-AE). Second, after adding one to each SpC and Log_2_-transformation (columns AF-AK), we calculated the average Log_2_-transformed, normalized SpC for both the Kir2.1^WT^ and Kir2.1^Δ314-315^ BioID experiments (columns AL and AM). The ratio *R* was then calculated by performing a power of 2 transformation (column AN). The statistical significances of the difference between the Kir2.1^WT^ and Kir2.1^Δ314-315^ BioID experiments were computed using a two-sample *t* test on the Log_2_-transformed data (column AO). Kir2.1 BioID hits with both *R* > 2 and *p* < 0.05 were classified as Kir2.1^WT^-preferred interactors whereas proteins with both *R* < 0.5 and *p* < 0.05, *i.e.* a 2-fold decrease, were classified as Kir2.1^Δ314-315^-mutant preferred interactors (column AP). A permutation analysis was performed to estimate the false discovery rate (*FDR* < 0.007).

##### Immunofluorescence (IF) Staining Experiments

IF staining experiments were performed utilizing either HEK293 cells or freshly isolated rat ventricular myocytes, as previously described (15) using the following primary and secondary antibodies: PKP4 (ThermoFisher Scientific, PA5-66855, dilution 1/500), Kir2.1 (Alomone Labs, Jerusalem, Israel, AGP-044, dilution 1/20), Actinin (Sigma, SAB4503474, 1/300), GM130 (Novus Biologicals, NBP2-53420; dilution 1/200), FLAG (Sigma-Aldrich; F3165, dilution: 1/50), Alexa Fluor 488 donkey anti-mouse, Alexa Fluor 594 donkey anti-guinea pig and Alexa Fluor 647 donkey anti-rabbit (Jackson ImmunoResearch, West Grove, PA, dilution 1/400).

##### Patch-Clamping Experiments

HEK293 cells were grown in 60-mm dishes and, upon reaching ∼60–70% confluence, were transiently transfected using X-tremeGENE HP DNA transfection reagent (Sigma, 6366244001) following the supplier's directions. The DNA plasmids used in the patch-clamp experiments were: CRISPR/Cas9 construct pX459-gPKP4 and negative control pX459 and protein expression vectors pcDNA3-HA-PKP4 and “empty vector” negative control pcDNA3-HA. A GFP-expression vector was also co-transfected to visualize the transfected cells during the patch-clamp experiments. Patch-clamp experiments were performed 48 h after transfection, as previously described (12, 13, 15). Inward rectifier potassium currents (I_Kir2.1_) were recorded at room temperature (21–23 °C) using the whole-cell patch-clamp technique and filtered at half the sampling frequency (12, 13, 15). Series resistance was compensated manually and ≥80% compensation was achieved. Under our experimental conditions, no significant voltage errors (<5 mV) because of series resistance were expected with the micropipettes used.

##### Experimental Design and Statistical Rationale

The total number of samples analyzed and the statistical tests used in the various experiments are indicated throughout the text, *e.g.* triplicate BioID experiments were performed, the number of transfected cell batches (*N*) and number of cells (*n*) used in the patch-clamping experiments are shown, or the statistical significances of the difference between the Kir2.1^WT^ and Kir2.1^Δ314-315^ BioID experiments were computed using a two-sample *t* test on the Log_2_-transformed data.

## RESULTS AND DISCUSSION

##### Kir2.1 Interactome Mapping Using BioID

**Fig. 1:**
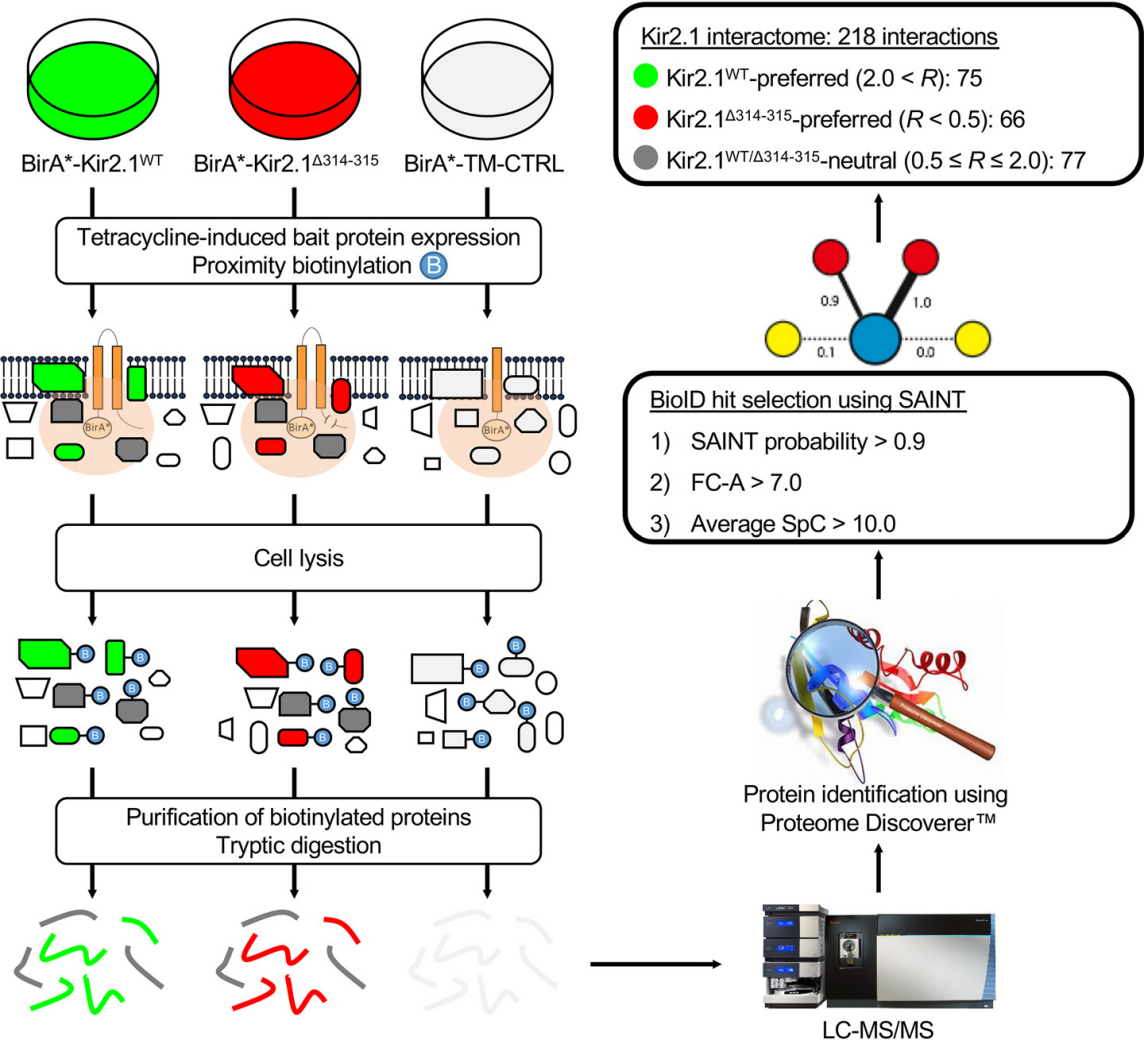
**Overall procedure to generate the Kir2.1 BioID interactome map.** Stable cells expressing BirA*-tagged Kir2.1^WT^, Kir2.1^Δ314-315^ or TM-CTRL bait proteins were generated using the Flp-In T-Rex 293 cell line. Expression of the bait proteins was induced by tetracycline and cells were treated with Supplemental biotin for 24 h. After cell lysis, biotinylated proteins were purified on streptavidin-agarose beads and digested with trypsin. Tryptic peptides were analyzed using LC–MS/MS and proteins were identified using Proteome Discoverer. After applying a stringent set of criteria, we identified 218 high-confidence Kir2.1 BioID hits. Using the normalized Kir2.1^WT/Δ314-315^ SpC ratio “*R* ”, we classified the interactors in three categories: 75 Kir2.1^WT^-preferred interactors, 66 Kir2.1^Δ314-315^-preferred interactors and 77 Kir2.1^WT/Δ314-315^-neutral interactors. CTRL: control; LC–MS/MS: liquid chromatography with tandem MS; SAINT: Significance Analysis of INTeractome; SpC: spectral counts; TM: transmembrane; WT: WT.

Using the proximity-labeling technology BioID (16, 17, 18), we generated a map of the Kir2.1 interactome (overall procedure is described in Fig. 1). Both Kir2.1^WT^ and Kir2.1^Δ314-315^, an ATS-associated mutant protein for which Golgi export is impaired (4, 19, 20), were used as BirA*-tagged BioID baits. We performed patch-clamping analyses of both the BirA*-tagged Kir2.1^WT^ and Kir2.1^Δ314-315^ bait proteins (supplemental Fig. S2). As expected, HEK293 cells expressing BirA*-tagged Kir2.1^WT^, but not mutant BirA*-tagged Kir2.1^Δ314-315^ cells, exhibited the characteristic ability to strongly rectify, that is to pass K^+^ current in the inward direction much more readily than outward. Moreover, BirA*-tagged Kir2.1^WT^ channels maintain their sensitivity to Ba^2+^ blocking (supplemental Fig. S2). As a negative control, we used the TM-CTRL construct, a yeast transmembrane domain fused to the BioID BirA* tag. We investigated the subcellular localization of both Kir2.1^WT^ and Kir2.1^Δ314-315^ bait proteins as well as the TM-CTRL by IF staining (supplemental Fig. S3). After expression of the BirA*-Kir2.1^WT^, BirA*-Kir2.1^Δ314-315^ and BirA*-TM-CTRL bait proteins in HEK293 cells (supplemental Fig. S4) and purification of the biotinylated proteins on streptavidin-agarose beads, the purified protein extracts were subjected to MS/MS analysis for identification. The Kir2.1^WT^, Kir2.1^Δ314-315^ and TM-CTRL baits were each assessed in triplicate BioID experiments. A stringent set of criteria were used for the selection of the Kir2.1 BioID hits: i) average SpC in either the Kir2.1^WT^ or the Kir2.1^Δ314-315^ BioID experiments > 10; ii) SAINT probability score (27, 28) > 0.9; and iii) primary fold change FC-A score (28) >7. Our proteomic screen resulted in the identification of 218 high-confidence Kir2.1 BioID hits, the vast majority of which are novel putative Kir2.1 direct or indirect interactors (supplemental Table S3). Interestingly, some of the newly identified BioID interactors include products of genes previously associated with physiological or pathophysiological function of the myocardium, *e.g.* UTRN (29, 30) and PKP4 (31, 32), as described in detail below. Curation of the literature and of the functional data reported in PubMed for each of these genes led to the identification of a dozen of known protein complexes or functionally related biological modules in our list of 218 high-confidence Kir2.1 BioID hits, *e.g.* the cadherin adhesome and the COPII complex.

To assess the sensitivity of our BioID proteomic screen, we measured the proportion of the previously described Kir2.1 interactors that are also present in our list of 218 high-confidence Kir2.1 BioID interactors. A review of several protein interaction repositories [BioGRID (33), IntAct (34), HPRD (35), HuRI (36, 37)] identified 24 putative Kir2.1 protein interactions. After literature curation, we selected 16 high-confidence Kir2.1 interactions that had been observed in at least two experiments and/or validated for their functional relevance (supplemental Table S1). We note, though, that some of these interactions have been studied using the *Mus musculus* or *Rattus norvegicus* proteins, but not the human proteins (columns I–J in supplemental Table S1). Out of all the 24 previously reported putative Kir2.1 interactors, 10 (∼42%) were identified in our BioID screen. Similarly, out of the 16 high-confidence Kir2.1 interactions, 7 (∼44%) were identified in our BioID screen, *i.e.* SAP97, CASK, CAV1, LIN7C, AKAP5, Kir2.6 and Kir2.3 (supplemental Table S1 and S3). This analysis suggests a high sensitivity of >40% for our BioID assay. By comparison, the sensitivity of high-throughput protein interaction mapping assays used on a proteome-scale ranges from ∼20 to ∼35% (38).

##### Kir2.1^WT^ versus Kir2.1^Δ314-315^ Interactome Profiling

**Fig. 2:**
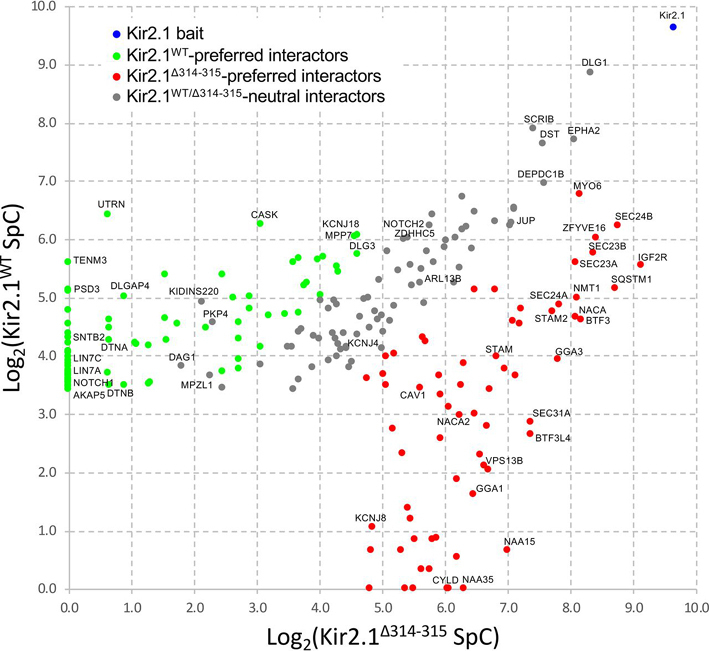
**Kir2.1^WT^*versus* Kir2.1^Δ314-315^ interactome profiling.** Variations in the Kir2.1^WT^ and Kir2.1^Δ314-315^ interactome profiles are visualized in a scatter plot representing the average Log_2_-transformed, normalized SpC counts for both the Kir2.1^WT^ (*y* axis) and Kir2.1^Δ314-315^ (*x* axis) bait proteins. The Kir2.1 bait, Kir2.1^WT^-preferred interactors, Kir2.1^Δ314-315^-preferred interactors and Kir2.1^WT/Δ314-315^-neutral interactors are represented as blue, green, red and gray dots, respectively.

Understanding the molecular mechanisms that govern the regulation of inward rectifier potassium currents by Kir2.1 in both normal and disease contexts should help uncover novel targets for therapeutic intervention in ATS1 and other Kir2.1-associated channelopathies. Beyond interaction mapping, our proteomic experiments also aims at capturing the differences between the interaction profiles of Kir2.1^WT^ and Kir2.1^Δ314-315^. Using the normalized Kir2.1^WT/Δ314-315^ SpC ratio “*R*” (supplemental Table S3, columns AN-AP; see Methods for details about the calculation, normalization and statistical analysis), we classified the 218 high-confidence Kir2.1 BioID hits: 75 tend to interact preferentially with Kir2.1^WT^, 66 interact preferentially with Kir2.1^Δ314-315^ and the remaining 77 BioID interactors interact with both the Kir2.1^WT^ and Kir2.1^Δ314-315^ proteins (*FDR* < 0.007; Fig. 2). For example, the normalized SpC counts for UTRN in the BioID experiments are: 86.0 SpC for Kir2.1^WT^ and 0.9 SpC for Kir2.1^Δ314-315^, *i.e. r =* 56.0 and *p* = 8 × 10^−4^. UTRN is thus classified as a Kir2.1^WT^-preferred interactor.

The ATS1-associated mutation Kir2.1^Δ314-315^ blocks Kir2.1 Golgi export (4, 19, 20). Hence, Kir2.1^WT^-preferred interactors may encompass both: i) protein interactors that are directly involved in the normal trafficking of Kir2.1, and ii) protein interactors that, as an indirect consequence of the Golgi-trapping, do not interact with Kir2.1^Δ314-315^ simply because it is not present at the plasma membrane. Interestingly, out of the 75 Kir2.1^WT^-preferred interactors, 13 of them have been associated (mutation and/or GWAS hit) with one or several heart-related traits or diseases, *e.g.* atrial fibrillation (AF) or systolic/diastolic blood pressure (supplemental Table S3, column AR).

Most ATS mutants, including Kir2.1^Δ314-315^, exert a dominant-negative effect on Kir2.1^WT^ channels, as oppose to an haploinsufficiency effect (20). Accordingly, protein interactions involving Kir2.1^Δ314-315^-preferred interactors may also encompass molecular mechanisms that are not properly regulated in the presence of Kir2.1^Δ314-315^. For example, the Kir2.1^Δ314-315^ mutation may result in the gain of an interaction (or increased binding affinity) between Kir2.1^Δ314-315^ and a protein present in the Golgi that perturbs normal trafficking and results in the trapping of Kir2.1^Δ314-315^. On one hand, the increased ability for Kir2.1^Δ314-315^ to interact with some of these Kir2.1^Δ314-315^-mutant preferred interactors may be an indirect consequence of the Δ314-315 mutation and these Kir2.1^Δ314-315^-mutant preferred interactors are thus likely to have low relevance to the disease. On the other hand, the increased ability for Kir2.1^Δ314-315^ to interact with some of these Kir2.1^Δ314-315^-mutant preferred interactors may be a direct consequence of the Δ314-315 mutation and some of these Kir2.1^Δ314-315^-mutant preferred interactors may thus be possible direct contributing factors to the disease. Interestingly, out of the 66 Kir2.1^Δ314-315^-preferred interactors, 10 of them have been associated (mutation and/or GWAS hit) with one or several heart-related traits or diseases, *e.g.* atrial fibrillation (AF) or systolic/diastolic blood pressure (supplemental Table S3, column AR). Below, we discuss in details the potential implications associated with some of these Kir2.1^WT^-preferred interactors, *e.g.* UTRN, and Kir2.1^Δ314-315^-preferred interactors, *e.g.* NAC and IGF1R.

**Fig. 3:**
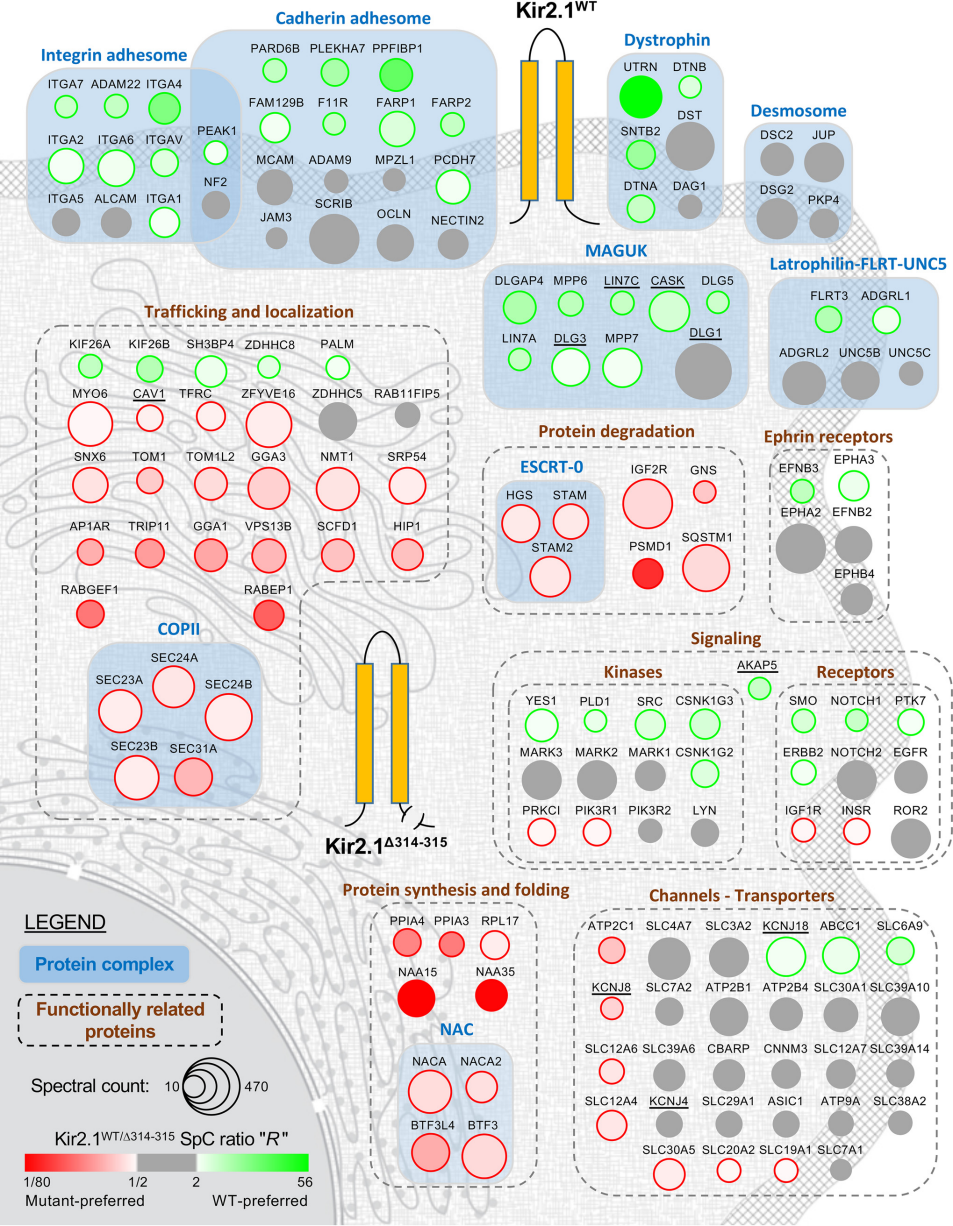
**Graphical representation of the Kir2.1 BioID interactome.** Protein complexes and groups of functionally related proteins encompassing 152 out of the 218 high-confidence Kir2.1 BioID hits are depicted in a cell. Major organelles in the cell (nucleus, endoplasmic reticulum, Golgi apparatus and cytoplasmic membrane) are shown (light gray) in the background to roughly indicate the approximate subcellular localization of the proteins in the cell. The Kir2.1^WT^-preferred interactors, Kir2.1^Δ314-315^-preferred interactors and Kir2.1^WT/Δ314-315^-neutral interactors are represented as green, red and gray circles, respectively. The color intensity of each circle is an indicator of the strength of the normalized Kir2.1^WT/Δ314-315^ SpC ratio “*R*” value. The size of each circle represents the average SpC counts observed in either the Kir2.1^WT^ or Kir2.1^Δ314-315^ BioID experiment (whichever is the largest is represented in the figure).

The analysis of the variations in the interactome profiles of Kir2.1^WT^ and Kir2.1^Δ314-315^ allows us to identify protein complexes or groups of functionally related proteins that, for the most part, tend to interact primarily with either Kir2.1^WT^ or Kir2.1^Δ314-315^ (Fig. 3). For example, most of the proteins in the MAGUK complex interact preferentially with Kir2.1^WT^ (colored in green in Fig. 3). In contrast, Kir2.1 interactors in the Nascent polypeptide-associated complex (NAC) bind preferentially to Kir2.1^Δ314-315^ (colored in red in Fig. 3). Interestingly, the “Channels-Transporters” group encompasses proteins that interact preferentially with either cytoplasmic membrane-bound Kir2.1^WT^, Golgi-trapped Kir2.1^Δ314-315^ or that are Kir2.1^WT/Δ314-315^-neutral interactors (colored in gray in Fig. 3). This observation indicates that Kir2.1 not only interacts with other channels-transporters at the cytoplasmic membrane but that the co-trafficking of these proteins in the endoplasmic reticulum (ER) and Golgi apparatus is an important aspect of their function. Thus, the previously described dynamic reciprocity for the regulation of Na_v_1.5 sodium and Kir2.1 potassium channel expression, which controls cardiac excitability and arrhythmia (12), likely extends to other channelosome protein complexes beyond Na_v_1.5.

##### Enrichment Analyses

To further extend our analysis of the protein complexes and the groups of genes that interact with Kir2.1, we performed a gene set enrichment analysis using DAVID (39) (supplemental Table S4). We observed that Kir2.1^WT^-preferred interactors are enriched for protein families involved in cell adhesion [GO:0007155, Fold Enrichment (FE) = 8, *p* = 2 × 10^−8^], including integrin complex proteins (GO:0008305, FE = 55, *p* = 6 × 10^−8^) and cadherin adhesome proteins (GO:0098641, FE = 6.1, *p* = 9 × 10^−4^). In addition to the protein domains characteristic of integrin proteins, *e.g.* InterPro domain IPR018184 (FE = 94, *p* = 3 × 10^−9^), Kir2.1^WT^-preferred interactors also tend to contain PDZ domains (IPR001478, FE = 15, *p* = 2 × 10^−7^), further highlighting the previously described key role of PDZ domain-scaffolding proteins in the modulation of Kir2.1 channelosomes (8). As expected, top KEGG pathways enriched in Kir2.1^WT^-preferred interactors include arrhythmogenic right ventricular cardiomyopathy (hsa05412), dilated cardiomyopathy (hsa05414) and hypertrophic cardiomyopathy (hsa05410) (FE > 17, *p* < 2 × 10^−5^).

In contrast, Kir2.1^Δ314-315^-preferred interactors are enriched for protein families involved in many aspects of intracellular protein transport (GO:0006886, FE = 16, *p* = 3 × 10^−12^), including COPII (GO:0030127, FE = 145, *p* = 3 × 10^−8^) and ER to Golgi transport (GO:0012507, FE = 22, *p* = 7 × 10^−4^). On the same note, seven proteins containing the multipurpose docking adapter VHS domain, *e.g.* TOM1 and STAM, which are associated with vesicular trafficking, are enriched among the Kir2.1^Δ314-315^-preferred interactors (IPR002014, FE = 222, *p* = 1 × 10^−13^). Thus, in agreement with the previous reports (4, 19, 20), our data show that ATS1 pathogenesis associated with the Kir2.1^Δ314-315^ mutation underlies malfunctions in the mechanisms governing Kir2.1 trafficking from the Golgi to the cytoplasmic membrane.

Kir2.1^Δ314-315^-preferred interactors are also linked to ESCRT-0 (GO:0033565, FE = 289, *p* = 3 × 10^−5^) and lysosome (hsa04142, FE = 7, *p* = 0.02), suggesting that this ATS1 mutation leads to increased targeting of Kir2.1 to lysosomal degradation. These observations agree with the previous report by Kolb *et al.* that Kir2.1 degradation is primarily lysosomal dependent and requires ESCRT (40). However, using a pharmacological approach to inhibit either proteasomal or vacuolar proteases, Kolb *et al.* also observed that Kir2.1^Δ314-315^ mutation targets the channel for proteasomal degradation rather than vacuolar-dependent degradation (40). Though we indeed note the presence of PSMD1, the 26S proteasome nonATPase regulatory subunit 1, among the Kir2.1^Δ314-315^-preferred interactors, our enrichment analysis does not provide support to the “proteasome hypothesis” but instead, strongly suggests that vacuolar-dependent degradation remains an important route responsible for the increased Kir2.1^Δ314-315^ protein turnover.

Strikingly, all the aforementioned functional attributes and the vast majority of the gene sets are specifically enriched in either Kir2.1^WT^-preferred interactors or Kir2.1^Δ314-315^-preferred interactors, but not both. For example, out of the top 30 gene sets enriched for Kir2.1^WT^-preferred interactors (*p* < 1 × 10^−3^), only one was also enriched in the Kir2.1^Δ314-315^-preferred interactors: basolateral plasma membrane (GO:0033565, FE = 6, *p* = 0.02) (supplemental Table S5). Similarly, out of the top 34 gene sets enriched for Kir2.1^Δ314-315^-preferred interactors (*p* < 1 × 10^−3^), only two were also enriched in the Kir2.1^WT^-preferred interactors (supplemental Table S5). This observation further underscores the fact that the Kir2.1^WT^ and Kir2.1^Δ314-315^ proteins obey to dramatically different fates in the cell.

##### Kir2.1 BioID Interactome: A Repository for Novel Biological Hypotheses

The Kir2.1 BioID interactome data set represents a repository for numerous, novel biological hypotheses for genes and molecular mechanisms implicated in Kir2.1-associated cardiomyopathies. For example, out of the 218 high-confidence Kir2.1 interactors identified in our BioID screen, 37 of them have been associated (mutation and/or GWAS hit) with one or several heart-related traits or diseases, *e.g.* atrial fibrillation (AF) or systolic/diastolic blood pressure (supplemental Table S3, column AR). Below, we describe a few examples: UTRN, NACA, IGF1R and PKP4.

*UTRN* was identified in our BioID screen as a Kir2.1^WT^-preferred interactor (normalized SpC counts: Kir2.1^WT^: 86.0 and Kir2.1^Δ314-315^: 0.9; supplemental Table S3). *Utrophin* deficiency worsens cardiac contractile dysfunction present in *dystrophin*-deficient mice (29). Interestingly, a race-stratified genome-wide gene-environment interaction association study recently identified polymorphic variations in the *UTRN* gene locus as potential risk factors in peripheral arterial disease (30). UTRN is part of the Dystrophin‐associated proteins (DAP) complex (41) and several other DAP proteins interact with and modulate both sodium and potassium channelosome protein complexes (8, 11, 42). This novel Kir2.1-UTRN interaction further underlines the role of DAP in regulating these complexes and malfunction of these molecular mechanisms may contribute to pathophysiological conditions of the myocardium.

##### Nascent-Polypeptide-Associated Complex (NAC)

Our BioID screen identified four NAC-associated proteins as Kir2.1^Δ314-315^-preferred interactors, *i.e.* NACA, NACA2, BTF3 and BTF3L4. For example, the normalized SpC counts for NACA are as follows: Kir2.1^WT^: 24.4 and Kir2.1^Δ314-315^: 270.7 (supplemental Table S3). The heterodimeric NAC protein complex binds to newly synthesized polypeptide chains that lack a signal peptide motif as they emerge from the ribosome, blocking interaction with the signal recognition particle (SRP) and preventing mistranslocation to the endoplasmic reticulum (43). Potential roles for NAC complex thus range from protein folding chaperone to negative regulator of translocation into the endoplasmic reticulum (43). We note, though, that NACA has also been described as a transcription factor (44), *e.g.* in collaboration with the heart-specific HDAC-dependent repressor SMYD1 (45). The roles of NACA and NAC in the cell remain to be comprehensively characterized.

Several observations link NACA to heart biology and diseases. For example, GWAS studies have identified *NACA* gene variants as potential risk factors in atrial fibrillation (AF) (46, 47), or as a regulator of myocardial mass (48). In both *Drosophila melanogaster* flies and *Danio rerio* zebrafish, the knock-down of *NACA*, *i.e.* fly *NAC*α and zebrafish *skNAC,* results in severe cardiac muscle defects (48, 49). In mouse, *Naca* deficiency results in embryonic lethality with cardiac developmental defects (50). Park *et al.* originally linked this phenotype to the role of NACA as a transcription factor and as a major binding partner for SMYD1 in the developing heart (50). The Kir2.1-NACA interaction suggests a more complex role for NACA in the myocardium. Indeed, Kir2.1 deficiency results not only in the loss of inward rectifier potassium currents but also in developmental defects and dysmorphic features, *e.g.* low-set ears, wide-set eyes, small mandible, clinodactyly and syndactyly (4, 5, 51, 52, 53). The molecular mechanism underlying these dysmorphic features is poorly understood. The Kir2.1-NACA interaction provides a biophysical bridge between the respective roles of these two proteins during biological development in general and cardiomyogenesis in particular.

##### Cross-talks between Kir2.1 and Signal Transduction Pathways

The Kir2.1 interactor data set as a whole is enriched for proteins involved in various aspects of signal transduction, *i.e.* enrichment is observed for all three classes of Kir2.1 interactors (WT, mutant and neutral; GO:0007165, FE > 2.4, *p* < 0.01). Each class of interactors is enriched with different signaling pathways, though. Examples of signaling pathways over-represented in our Kir2.1^WT^-preferred interactor data set include the ephrin receptor signaling pathway (GO:0048013, FE = 12, *p* = 5 × 10^−3^) and the Hedgehog signaling pathway (KEGG hsa04340, FE = 26, *p* = 5 × 10^−3^).

Likewise, the Kir2.1^Δ314-315^-preferred interactors are enriched for proteins involved in the insulin-like growth factor receptor signaling pathway, *e.g.* IGF1R and INSR (GO:0048009, FE = 57, *p* = 1 × 10^−3^). The normalized SpC counts for IGF1R and INSR are as follows: Kir2.1^WT^: 12.1 and 15.7, and for Kir2.1^Δ314-315^: 31.6 and 35.4, respectively (supplemental Table S3). GWAS studies have identified *IGF1R* gene variants as potential risk factors in atrial fibrillation (AF) (46, 47), as well as a regulator of myocardial mass (48) and global electrical heterogeneity ECG (54). We hypothesize that the Kir2.1-IGF1R interaction is part of a molecular mechanism that underlies the functional associations between IGF1R and cardiac physiology.

Could the Kir2.1-IGF1R interaction also help better understand the potential role of Kir2.1 in diabetes? A functional link between Kir2.1 and the insulin-like growth factor receptor signaling pathway has been previously proposed. As early as 1998, Wischmeyer *et al.* observed that treatment of *Xenopus* oocytes with insulin was found to robustly suppress I_Kir2.1_ (55). Electrical activity plays a central role in glucose-stimulated insulin secretion from human pancreatic β-cells. We note that Kir2.1 is expressed in human pancreatic islets (56) and that functional Kir2.1 currents are present in human β-cells (57). Riz *et al.* predicted that blocking Kir2.1 channels increases the rate of insulin secretion and that hyperactive Kir2.1 channels may lead to reduced insulin secretion (57). Similarly, in a recent mathematical model, Kir2.1 was predicted to be a critical actor in the regulation of oscillations in cellular activity that underlie insulin pulsatility in pancreatic islets cells lacking K(ATP) channels (58). On the same note, several publications link abnormal QT interval regulation and diabetes (59, 60, 61). Moreover, patients with long QT syndrome associated to potassium channel deficiency, *e.g.* KCNQ1 mutants, suffer from over-secretion of insulin, hyperinsulinemia, and symptomatic hypoglycemia (62). Our BioID data suggest that these functional associations between Kir2.1 and the insulin pathway need not be indirect but may also be mediated by biophysical interactions between Kir2.1 channels and insulin pathway proteins.

##### Desmosome

Several desmosome-associated proteins interact with both Kir2.1^WT^ and Kir2.1^Δ314-315^, *e.g.* JUP, DSG2, DSC2 and PKP4 (supplemental Table S3). Desmosomes act as mechanical cell-cell adhesion junctions maintaining the structural integrity of the tissue. Mutations in the *JUP*, *DSG2* and *DSC2* genes have been associated with Arrhythmogenic Right Ventricular Cardiomyopathy (ARVC) (63). Actually, with ∼half of ARVC patients carrying a mutation in one of the genes encoding desmosomal proteins expressed in the heart, ARVC can be considered a disease of the desmosome (63). Mutations in the *JUP* genes have also been associated with Naxos disease, a diffuse nonepidermolytic palmoplantar keratoderma with wooly hair and cardiomyopathy (64). We also note that *JUP* mRNA is highly differentially expressed in AF (65).

##### Functional Validation of PKP4 by Immunofluorescence and Patch-Clamp Analyses

*PKP4* is one of the desmosome proteins identified in our BioID experiment as interacting with both Kir2.1^WT^ and Kir2.1^Δ314-315^ (normalized SpC counts: Kir2.1^WT^: 23.3 and Kir2.1^Δ314-315^: 7.6). PKP4 is a multifunctional armadillo protein coordinating cell adhesion with cytoskeletal organization that also has a role in vesicle transport processes (66). Though less frequent than the ones observed for the *JUP*, *DSG2* and *DSC2* genes, mutations in the *PKP4* gene have been observed in ARVC patients (31, 32). Similarly to many other Kir2.1 interactors (8), PKP4 carries a PDZ binding site at its carboxyl terminus. We hypothesize that the Kir2.1-PKP4 interaction contributes to the regulation of Kir2.1.

**Fig. 4:**
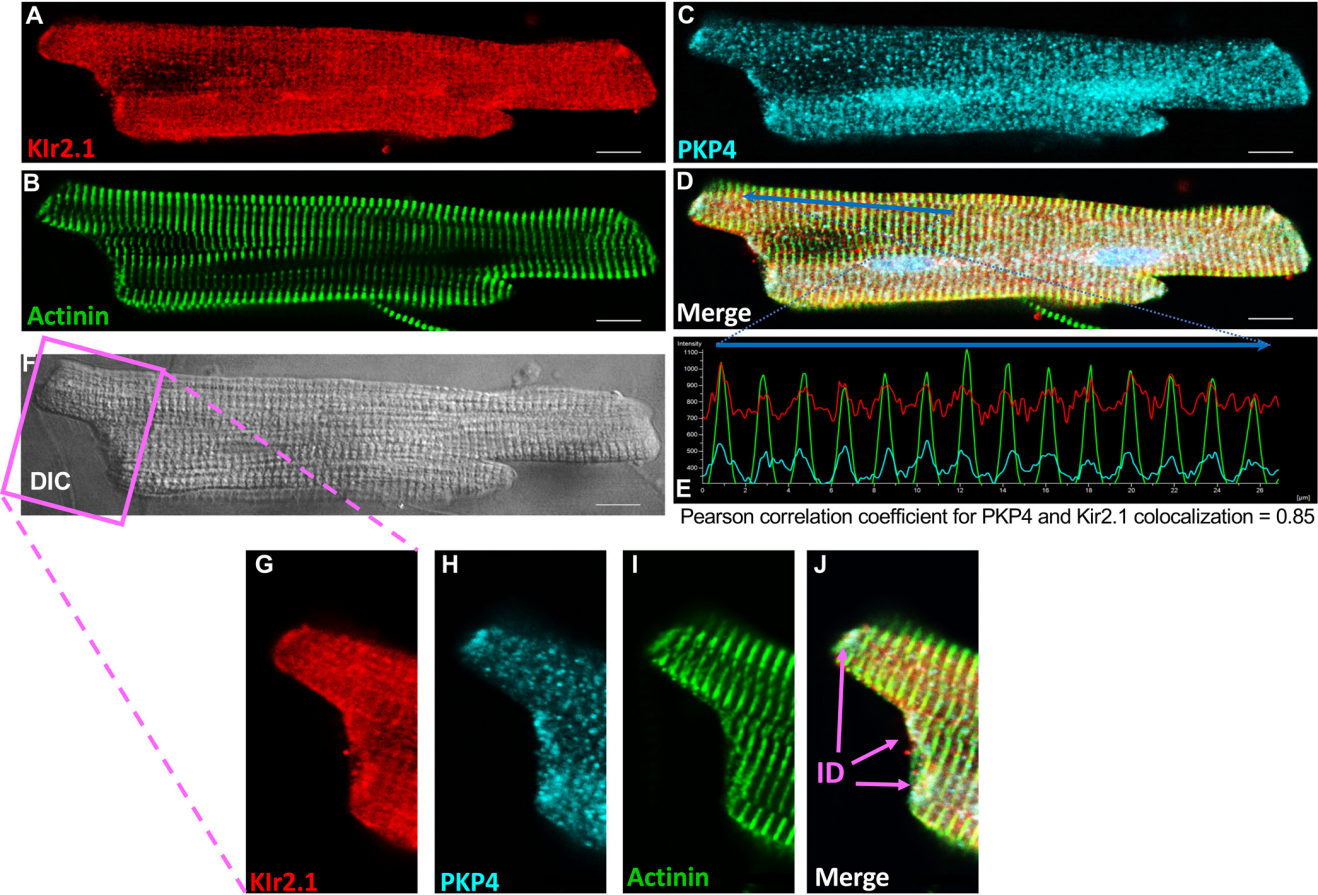
**Kir2.1 and PKP4 co-localize in adult ventricular myocytes.** Immunofluorescence (IF) staining analyses of the subcellular localization of Kir2.1 (*A*, *red*), Actinin (*B*, *green*) and PKP4 (*C*, light *blue*) in a freshly isolated rat adult ventricular myocytes. (*D*) Merge image. (*E*) Pixel intensity profile of PKP4, Kir2.1 and actinin along a line in the merge image, *i.e.* blue arrow shown in (*D*) showing the striated co-localization of the three proteins at the z-disks near the cardiac sarcomeres. (*F*) Differential interference contrast (DIC) image of the myocyte. (*G*–*J*) Zoomed in images of the intercalated disks. Scale bars: 10 μm. ID: intercalated disk.

As a first step toward validating the role of PKP4 as a regulator of Kir2.1, we investigated the subcellular localization of both proteins by immunofluorescence (IF) staining in freshly isolated adult rat ventricular myocytes. We observed that Kir2.1 and PKP4 co-localize at intercalated disks and at z-disks near the cardiac sarcomeres (Pearson correlation coefficient = 0.85) (Fig. 4).

**Fig. 5:**
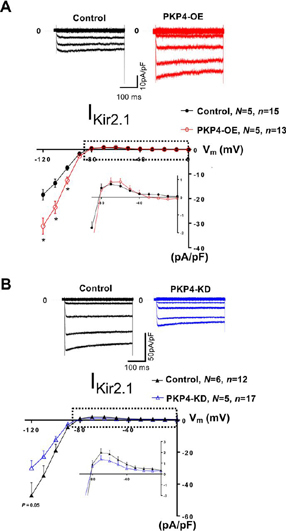
**PKP4 is a positive regulator of I_Kir2.1_.** Patch-clamping analyses in HEK293 cells upon genetic perturbation of *PKP4*, *i.e.* (*A*) upon overexpression of PKP4 and (*B*) upon CRISPR/Cas9-mediated depletion of PKP4. *P < 0.05 *versus* control. *N*: number of transfected cell batches; *n*: number of cells; OE: overexpression; *K_D_*: knock-down.

Finally, to assess the functional relevance of PKP4 to the modulation of I_Kir2.1_, we performed patch-clamp in the voltage clamp configuration (12, 13, 15) in a stable HEK293 cell line expressing Kir2.1 upon genetic perturbation of PKP4 (Fig. 5). Upon PKP4 overexpression, we observed a gain of I_Kir2.1_ density (Fig. 5*A*). Accordingly, upon CRISPR/Cas9-mediated depletion of PKP4, we observed a loss of I_Kir2.1_ density (Fig. 5*B*). Therefore, in addition to interacting with Kir2.1 (Fig. 2) and to being mutated in an ARVC patients (31, 32), PKP4 is a positive regulator of I_Kir2.1_ density.

## CONCLUSION

Evidence accumulated over the last 20 years strongly indicates that potassium channels function depends on a complex and dynamic ensemble of molecular events occurring in various organelles in the cell that help in the assembly and delivery of the right channel subunits to the right place in the cell membrane at the right time. Understanding such molecular events should help uncover novel insights into how channelosome proteins and accessory proteins participate in the control of cardiac excitability and the mechanisms of arrhythmias. Using a BioID proteomic approach, we have generated the most comprehensive Kir2.1 interactome map known to date with 218 high-confidence Kir2.1 BioID hits, the vast majority of which are novel putative Kir2.1 interactors. Moreover, our approach allowed us to identify novel proteins that interact preferentially with either Kir2.1^WT^ or the ATS1 mutant Kir2.1^Δ314-315^, thus uncovering novel molecular mechanisms underlying Kir2.1 function/dysfunction in ATS1. Our Kir2.1 interactome map represents a repository for numerous novel biological hypotheses. Using patch-clamping, we verified the functional relevance of one of our hits, PKP4, to the modulation of I_Kir2.1_, thus providing a molecular mechanism by which PKP4 may be involved in ARVC. This result validates the power of our BioID interactome approach in identifying functionally relevant Kir2.1 interactors and modulators of I_Kir2.1_.

## DATA AVAILABILITY

The MS raw data and Proteome Discoverer protein identification have been deposited to the ProteomeXchange Consortium via the PRIDE partner repository (26) with the data set identifier PXD011004.
