# Supplementary material for: Kir2.1 Interactome Mapping Uncovers PKP4 as a Modulator of the Kir2.1-Regulated Inward Rectifier Potassium Currents
Source: Mol Cell Proteomics. 2020 Nov 25;19(9):1436–49. doi: 10.1074/mcp.RA120.002071 (PMC8143648; doi:10.1074/mcp.RA120.002071)
Supplement: Supplementary file 1 [file mmc1.zip › mmc1/159891_0_supp_504458_q86317.pdf]

Supplemental Fig. S1

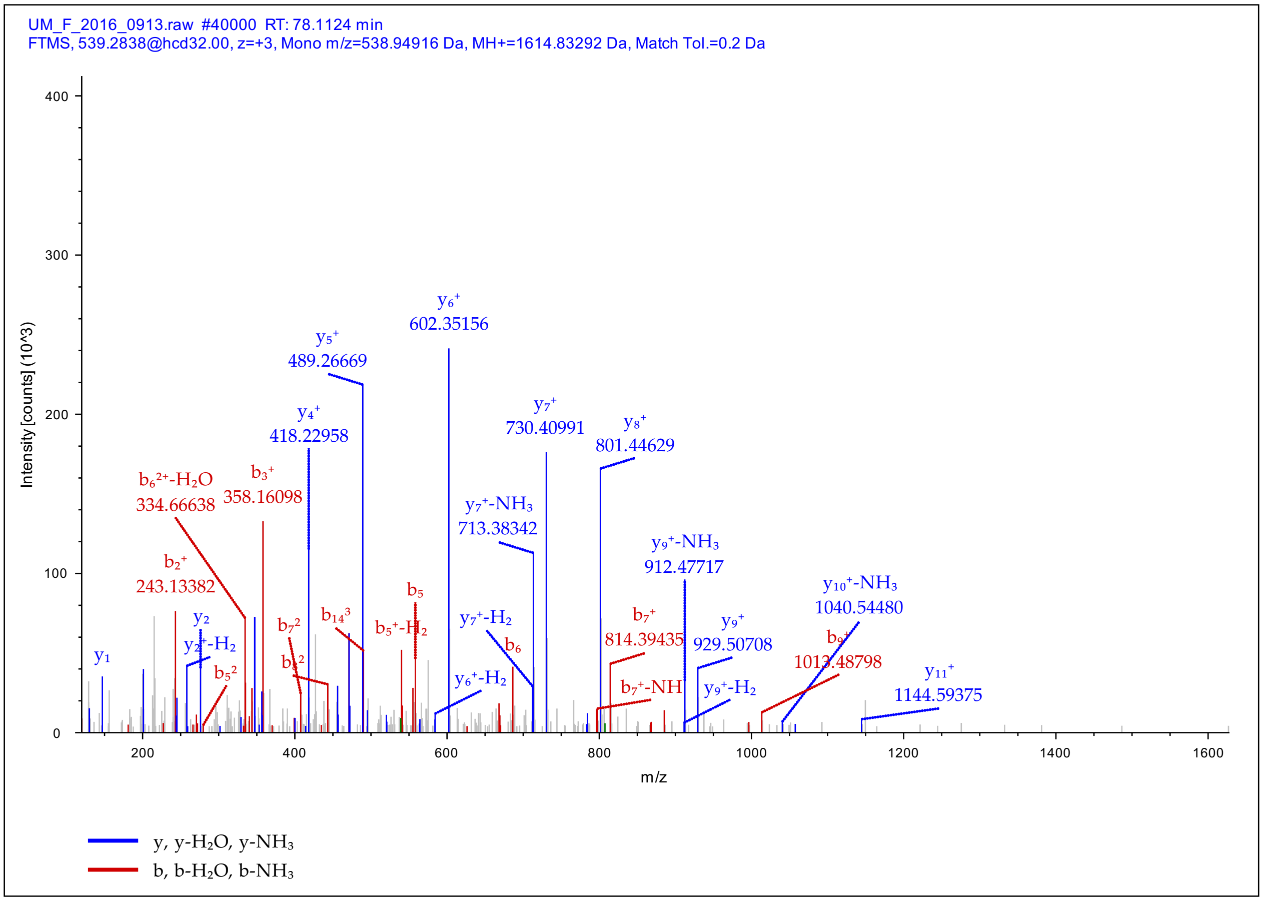

#1	b+	b2+	b3+	Seq.	y+	y2+	y3+	#2
1	114.09134	57.54931	38.70196	I				15
2	243.13393	122.07061	81.71616	Q-Deamid...	1501.74928	751.37828	501.25461	14
3	358.16088	179.58408	120.05848	D	1372.70668	686.85698	458.24041	13
4	471.24494	236.12611	157.75316	L	1257.67974	629.34351	419.89810	12
5	558.27697	279.64212	186.76384	S	1144.59568	572.80148	382.20341	11
6	686.33555	343.67141	229.45003	Q	1057.56365	529.28546	353.19273	10
7	814.39412	407.70070	272.13623	Q	929.50507	465.25617	310.50654	9
8	885.43124	443.21926	295.81526	A	801.44649	401.22689	267.82035	8
9	1013.48982	507.24855	338.50146	Q	730.40938	365.70833	244.14131	7
10	1126.57388	563.79058	376.19614	L	602.35080	301.67904	201.45512	6
11	1197.61099	599.30913	399.87518	A	489.26674	245.13701	163.76043	5
12	1268.64811	634.82769	423.55422	A	418.22962	209.61845	140.08139	4
13	1339.68522	670.34625	447.23326	A	347.19251	174.09989	116.40235	3
14	1468.72781	734.86755	490.24746	E	276.15540	138.58134	92.72332	2
15				K	147.11280	74.06004	49.70912	1

Supplemental Figure S1 | Peptide spectrum match identification details of a NACA2 peptide. Though a total of 110 spectral counts were measured for the NACA2 protein, it was identified with a single-peptide match. Here, we show a representative MS/MS spectrum of a Q2-deamidated peptide from NACA2 (128-142) with annotated fragments. The mother ion is +3 charged with monoisotopic m/z=538.95.

Supplemental Fig. S2

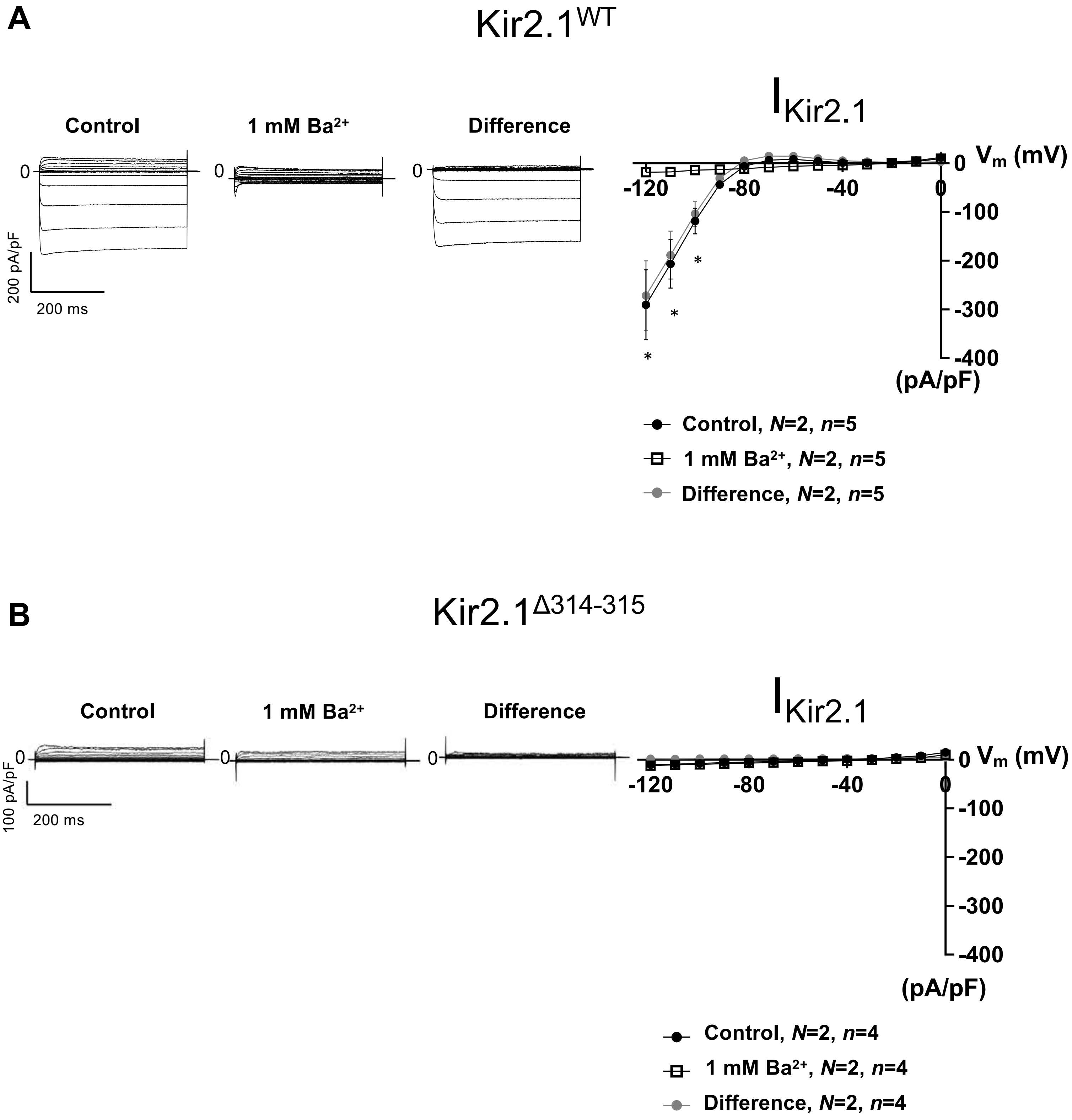

Supplemental Figure S2 | Patch-clamping analyses of the BirA*-FLAG tagged Kir2.1^{WT} or Kir2.1$^{\Delta 314\text{-}315}$ bait proteins. Current traces and I/V relationships from WT (**A**) or mutant (**B**) Kir2.1 are shown. *P<0.05 vs. control. N: number of transfected cell batches; n: number of cells.

Supplemental Fig. S3

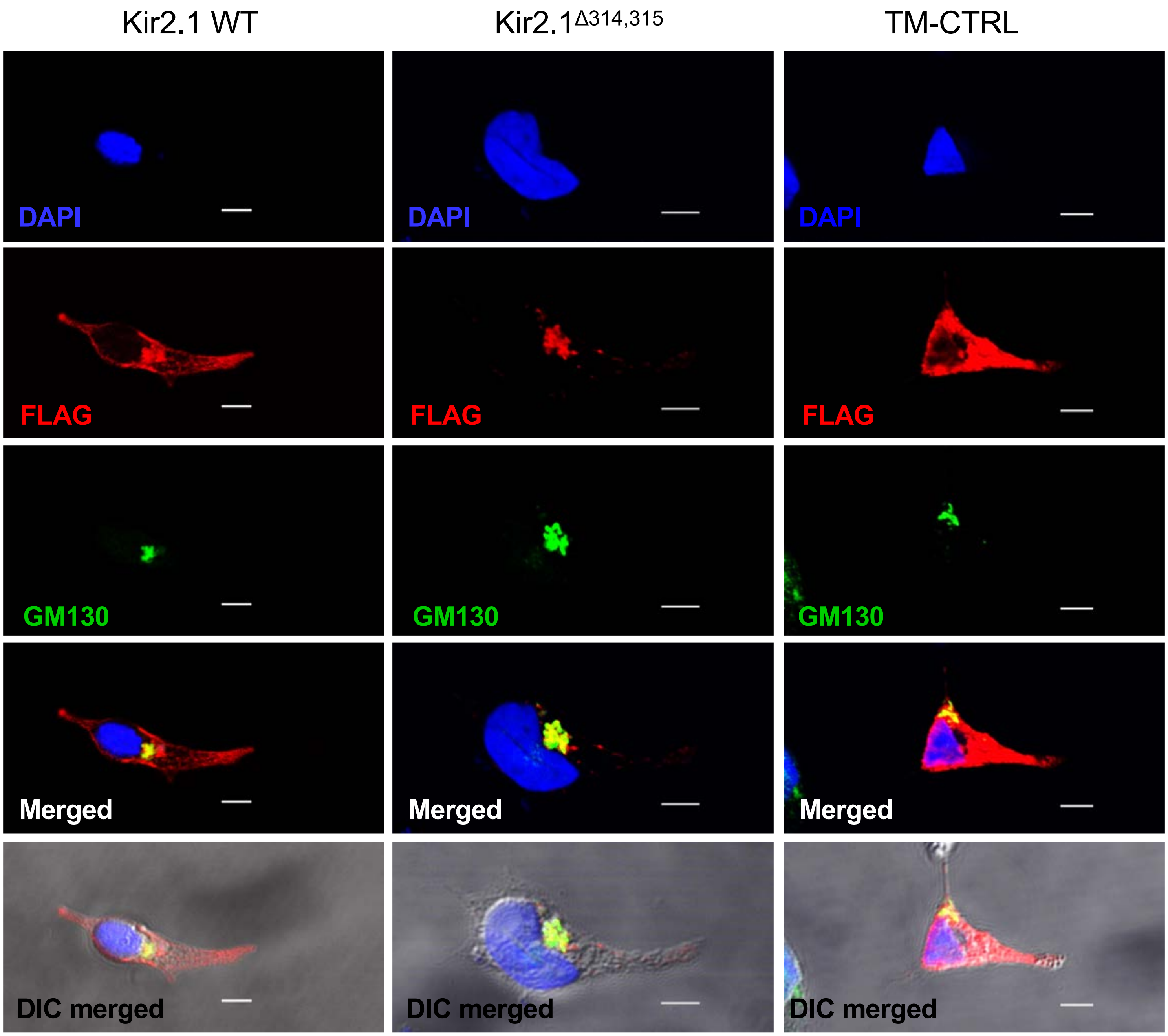

Supplemental Figure S3 | Immunofluorescence (IF) staining analyses of the subcellular localization of the BirA*-FLAG tagged Kir2.1^{WT}, Kir2.1$^{\Delta 314\text{-}315}$ or TM-CTRL bait proteins. GM130 was used as a marker for the Golgi apparatus and DAPI as a nuclear marker. Kir2.1^{WT} is detected at the plasma membrane, as well as in the cytoplasm, including the Golgi apparatus. Kir2.1$^{\Delta 314\text{-}315}$ is detected in the cytoplasm and overlaps almost exclusively with GM-130, in agreement with the previous reports of its trapping in the Golgi apparatus (Bendahhou et al, 2003, Ma et al, 2011, Plaster et al, 2001). TM-CTRL is detected at the plasma membrane as well as in the cytoplasm, including the Golgi apparatus. Scale bars: 10 µm.

Supplemental Fig. S4

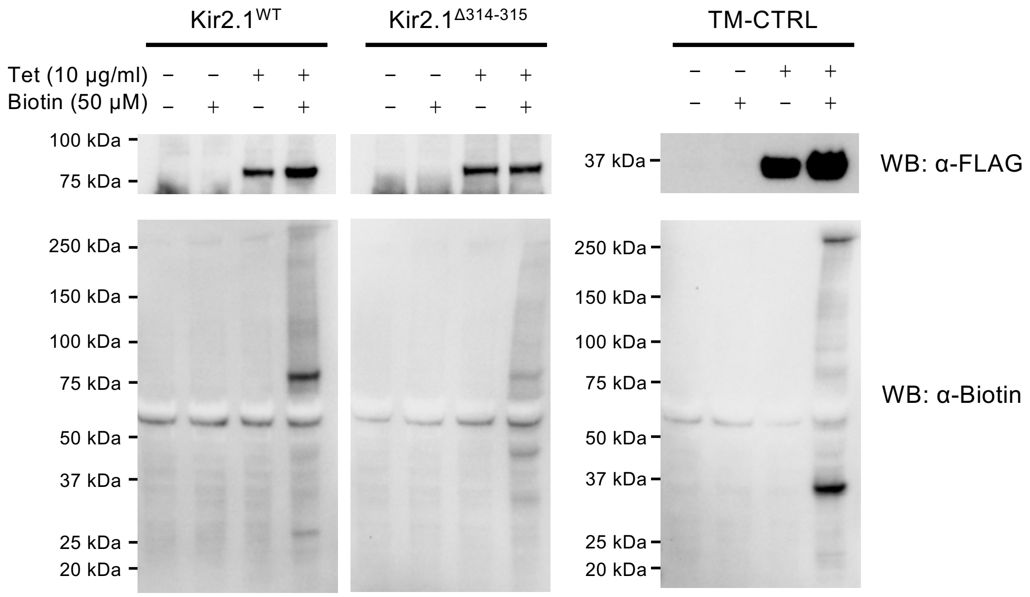

Supplemental Figure S4 | Western blot analyses of the BioID bait protein expression and biotinylated proteins. Expression of the BioID bait proteins was induced by tetracycline and cells were treated with supplemental biotin for 24 hours. Expression of the BirA*-FLAG tagged Kir2.1^{WT}, Kir2.1$^{\Delta 314\text{-}315}$ or TM-CTRL bait proteins is verified by Western blotting using FLAG antibody. The presence of the biotinylated proteins is assessed by Western blotting using α-biotin antibody. CTRL: control; Tet: tetracycline; TM: transmembrane; WB: Western blot; WT: wild type.
