# Supplementary material for: Kir2.1 Interactome Mapping Uncovers PKP4 as a Modulator of the Kir2.1-Regulated Inward Rectifier Potassium Currents
Source: Mol Cell Proteomics. 2020 Nov 25;19(9):1436–49. doi: 10.1074/mcp.RA120.002071 (PMC8143648; doi:10.1074/mcp.RA120.002071)
Supplement: Supplementary file 1 [file mmc1.zip › mmc1/159891_0_supp_504460_q86317.pdf]

## SUPPLEMENTAL SUPPLEMENT FILE

## SUPPLEMENTAL TABLE LEGENDS

**Supplemental Table S1 | List of the previously identified Kir2.1 interactors.** The table summarizes the list of the Kir2.1 protein interactors reported in the literature and public databases for which the report of a biophysical interaction assessed in at least one protein-protein interaction assay could be verified. The table lists various information, *e.g.* the source or the PubMed ID of the publication in which the interaction is reported, the type of assay used to test the interaction and the species of the proteins used to test the interaction. The interactions were classified as high-confidence (HC) if supported by multiple assays and/or functional data.

**Supplemental Table S2 | Sequences of the oligonucleotides used for cloning purposes and of the CRISPR/Cas9 gRNA constructs.**

**Supplemental Table S3 | BioID data and other information for the 218 high-confidence Kir2.1 interactors.** The table summarizes the BioID data for the 218 high-confidence Kir2.1 interactors including: columns H-P: spectral counts (SpC) for each protein in each of the $Kir2.1^{WT}$, $Kir2.1^{\Delta 314\text{-}315}$ or TM-CTRL triplicate BioID experiments; columns Q-S: average SpC; columns T-W: SAINT probability and FC-A scores (1, 2); columns X-AO: step-by-step description of the calculation of the normalized $Kir2.1^{WT/\Delta 314\text{-}315}$ SpC ratio "*R*" (see Methods for details); column AP: classification of the 75 $Kir2.1^{WT}$-preferred interactors, 66 $Kir2.1^{\Delta 314\text{-}315}$-preferred interactors and 77 $Kir2.1^{WT/\Delta 314\text{-}315}$-neutral interactors; column AQ: list of the Kir2.1 interactors previously identified in the literature; columns AR-AS: list of the Kir2.1 interactors for which functional links are previously described in the literature or in the NHGRI-EBI Catalog of published genome-wide association studies (default criteria were used: $P$ value for SNP/disease trait association $< 5 \times 10^{-8}$; release date: 2018-09-10) (3).

**Supplemental Table S4 | Summary of the gene set enrichment analyses.** The table contains two worksheets that summarize the findings of the gene set enrichment analysis for the 75 $Kir2.1^{WT}$-preferred interactors and the 66 $Kir2.1^{\Delta 314\text{-}315}$-preferred interactors using DAVID (4). We are showing the biological processes (8013 BP gene sets were tested), molecular functions (2645 MF gene sets were tested), cellular components (1264 CC gene sets were tested), KEGG pathway modules (298 KEGG gene sets were tested) and InterPro domain gene sets (4551 InterPro gene sets were tested) for which a $P$ value < 0.05 (Fisher exact test) was observed for the enrichment.

**Supplemental Table S5 | Most top gene set enrichment hits are specifically enriched in either $Kir2.1^{WT}$-preferred interactors or $Kir2.1^{\Delta 314\text{-}315}$-preferred interactors, but not both.** The table focuses on the top 30 gene set enrichments observed for the $Kir2.1^{WT}$-preferred interactors and the top 34 gene set enrichments observed for the $Kir2.1^{\Delta 314\text{-}315}$-preferred interactors ($P$ < 0.001). For comparison, gene counts and $P$ values are shown for the three classes of Kir2.1 interactors: $Kir2.1^{WT}$-preferred interactors, $Kir2.1^{\Delta 314\text{-}315}$-preferred interactors and 77 $Kir2.1^{WT/\Delta 314\text{-}315}$-neutral interactors.

## SUPPLEMENTAL REFERENCES

1. Choi, H., Larsen, B., Lin, Z. Y., Breitkreutz, A., Mellacheruvu, D., Fermin, D., Qin, Z. S., Tyers, M., Gingras, A. C., and Nesvizhskii, A. I. (2011) SAINT: probabilistic scoring of affinity purification-mass spectrometry data. *Nat Methods* 8, 70-73

2. Mellacheruvu, D., Wright, Z., Couzens, A. L., Lambert, J. P., St-Denis, N. A., Li, T., Miteva, Y. V., Hauri, S., Sardiu, M. E., Low, T. Y., Halim, V. A., Bagshaw, R. D., Hubner, N. C., Al-Hakim, A., Bouchard, A., Faubert, D., Fermin, D., Dunham, W. H., Goudreault, M., Lin, Z. Y., Badillo, B. G., Pawson, T., Durocher, D., Coulombe, B., Aebersold, R., Superti-Furga, G., Colinge, J., Heck, A. J., Choi, H., Gstaiger, M., Mohammed, S., Cristea, I. M., Bennett, K. L., Washburn, M. P., Raught, B., Ewing, R. M., Gingras, A. C., and Nesvizhskii, A. I. (2013) The CRAPome: a contaminant repository for affinity purification-mass spectrometry data. *Nat Methods* 10, 730-736

3. Morales, J., Welter, D., Bowler, E. H., Cerezo, M., Harris, L. W., McMahon, A. C., Hall, P., Junkins, H. A., Milano, A., Hastings, E., Malangone, C., Buniello, A., Burdett, T., Flicek, P., Parkinson, H., Cunningham, F., Hindorff, L. A., and MacArthur, J. A. L. (2018) A standardized framework for representation of ancestry data in genomics studies, with application to the NHGRI-EBI GWAS Catalog. *Genome Biol* 19, 21

4. Huang da, W., Sherman, B. T., and Lempicki, R. A. (2009) Systematic and integrative analysis of large gene lists using DAVID bioinformatics resources. *Nat Protoc* 4, 44-57
